# Gestational and postpartum maternal consequences of gestational diabetes mellitus

**DOI:** 10.3389/fendo.2026.1809244

**Published:** 2026-04-15

**Authors:** Chung-Kuan Wu, Chia-Yi Chang, Kok-Min Seow, Yen-Chun Huang, Chien-Wei Chuang, Mingchih Chen

**Affiliations:** 1 Division of Nephrology, Department of Internal Medicine, Shin-Kong Wu Ho-Su Memorial Hospital, Taipei, Taiwan; 2 School of Medicine, Fu-Jen Catholic University, New Taipei, Taiwan; 3 Division of Digital Informatics Management, Department of Digital Medicine, Shin Kong Wu Ho-Su Memorial Hospital, Taipei, Taiwan; 4 Department of Obstetrics and Gynecology, Cathay General Hospital, Taipei, Taiwan; 5 Department of Obstetrics and Gynecology, Shin-Kong Wu Ho-Su Memorial Hospital, Taipei, Taiwan; 6 Department of artificial intelligence, Tamkang University, New Taipei City, Taiwan; 7 AI Development Center, Fu Jen Catholic University, New Taipei City, Taiwan; 8 Graduate Institute of Business Administration, College of Management, Fu Jen Catholic University, New Taipei City, Taiwan

**Keywords:** chronic kidney disease, diabetes mellitus, gestational diabetes mellitus, gestational hypertension, preeclampsia, preterm labor

## Abstract

**Background:**

Gestational diabetes mellitus (GDM) is a significant complication during pregnancy with varying prevalence across countries and ethnicities. In Taiwan, although GDM prevalence rose from 7.6% to 13.4% between 2004 and 2015, its maternal gestational and extended consequences remained underexamined. The nationwide population-based study aims to investigate GDM-related risk factors and identify the critical period during which GDM likely poses long-term health risks.

**Methods:**

A total of 206,831 adult pregnant women from the National Health Insurance Research Database were divided into GDM (*n* = 8,204) and non-GDM (*n* = 198,627). After 1:1 matching of age and comorbidities, logistic and Cox regression was used to assess the odd and hazard ratio of maternal gestational and extended consequences of GDM. Kaplan-Meier analyses provided follow up events-free outcomes.

**Results:**

The incidence of preterm labor, preeclampsia, and gestational hypertension were significantly higher in the GDM group. The odd ratios of these consequences were 1.72, 2.86, and 2.85, respectively. GDM significantly affected the development of type 2 DM, chronic kidney disease (CKD), and ophthalmic disease. The hazard ratios of these diseases were 2.88, 1.54, and 1.63, respectively. Kaplan-Meier analysis revealed that GDM significantly increased these diseases during follow-up, especially within 2 years for type 2 DM and within 1 year for CKD and ophthalmic disease after delivery.

**Concolusion:**

GDM was associated with higher risks of preterm labor, gestational hypertension, preeclampsia, type 2 DM, CKD, and ophthalmic disease. Postpartum GDM follow-up time is 2 years for type-2 DM and 1 year for CKD and ophthalmic disease.

## Introduction

Gestational diabetes mellitus (GDM) is an important gestational complication during pregnancy. The prevalence of GDM in worldwide is approximated 14% of all pregnancies, and this value differs across different countries and ethnicities (1, 2). In Taiwan, the annual prevalence of GDM has increased by 1.8 folds from 7.6% to 13.4% between 2004 to 2015 (3).

During early pregnancy, insulin sensitivity is increased to promote the utility of glucose from metabolic adaptions for energy demands in the mother. Insulin resistance progresses while elevating human placental lactogen, estrogen and progesterone in the late second trimester. During the period, pancreatic beta cells can secrete more insulin in response to the physiologic change in healthy pregnant women. However, GDM occurs when pancreatic beta cells could not compensate for insulin resistance (4). Advanced maternal age, obesity, family history of diabetes and GDM in previous pregnancies are risk factors of GDM (5). In addition, women with existing gynecological diseases such as endometriosis and polycystic ovary syndrome (PCOS) are at risk of developing GDM (6, 7). Besides, pelvic inflammatory disease (PID) and uterine myoma can result in preterm birth (8, 9), and endometrial polyp is associated with potential risk of endometrial cancer (10).

Hyperglycemia during pregnancy is associated with adverse pregnancy outcomes such as pre-term birth, and gestational hypertension (11, 12). Maternal hyperglycemia leads to increased transplacental glucose transfer and causes fetal hyperglycemia, thus inducing excess levels of circulating insulin in fetus’ blood. Consequently, the status of hyperinsulinemia affects metabolism and accelerates the fetal growth, thus increasing the risk of macrosomia (13). The association of gestational hypertension and preeclampsia with GDM can be explained by the trophoblast inflammation and oxidative stress-induced maternal vasodilation dysfunction (14, 15).

In addition to the adverse outcome of GDM during pregnancy, women with GDM have at least a seven-fold increased risk of developing type 2 DM in the future compared with those without GDM (16). Limited studies have focused on the subsequent incidence of chronic kidney disease (CKD), cardiovascular disease, gynecologic malignancy, and ophthalmic disease after GDM (14–16). The effect period of GDM for these outcomes remains unknown. Therefore, firstly the nationwide population-based study aimed to investigate risk factors of GDM on the pregnant women including pre-term, preeclampsia, and gestational hypertension. Secondly, the nationwide population-based study investigated the possible extended consequences of GDM including type 2 DM, CKD, ophthalmic disease, cardiovascular disease, and malignancies. Finally, the nationwide population-based study was designed to detect the significant insulting period of GDM on extended consequences.

## Materials and methods

### Data sources and research samples

On March 1, 1995, Taiwan launched the National Health Insurance (NHI) program, in which more than 99% of the country’s total population was enrolled. The NHI system in Taiwan establishes the NHI Research Database and collects each patient’s individual detailed information on each health service. Information such as age, gender, cancer registry, operation, and disease diagnosis are included in this database. Complications of pregnancy, including preterm labor, gestational hypertension, and preeclampsia are recorded. Type 2 DM, CKD, ophthalmic disease, cardiovascular disease (CVD), and malignancies were included. The diseases are classified and numbered according to the International Classification of Diseases, 9th Revision, Clinical Modification (ICD-9-CM) and the International Classification of Diseases, 10th Revision (ICD-10). The codes were summarized in **Supplementary Table 1**.

### Study population and exclusion criteria

**Figure 1:**
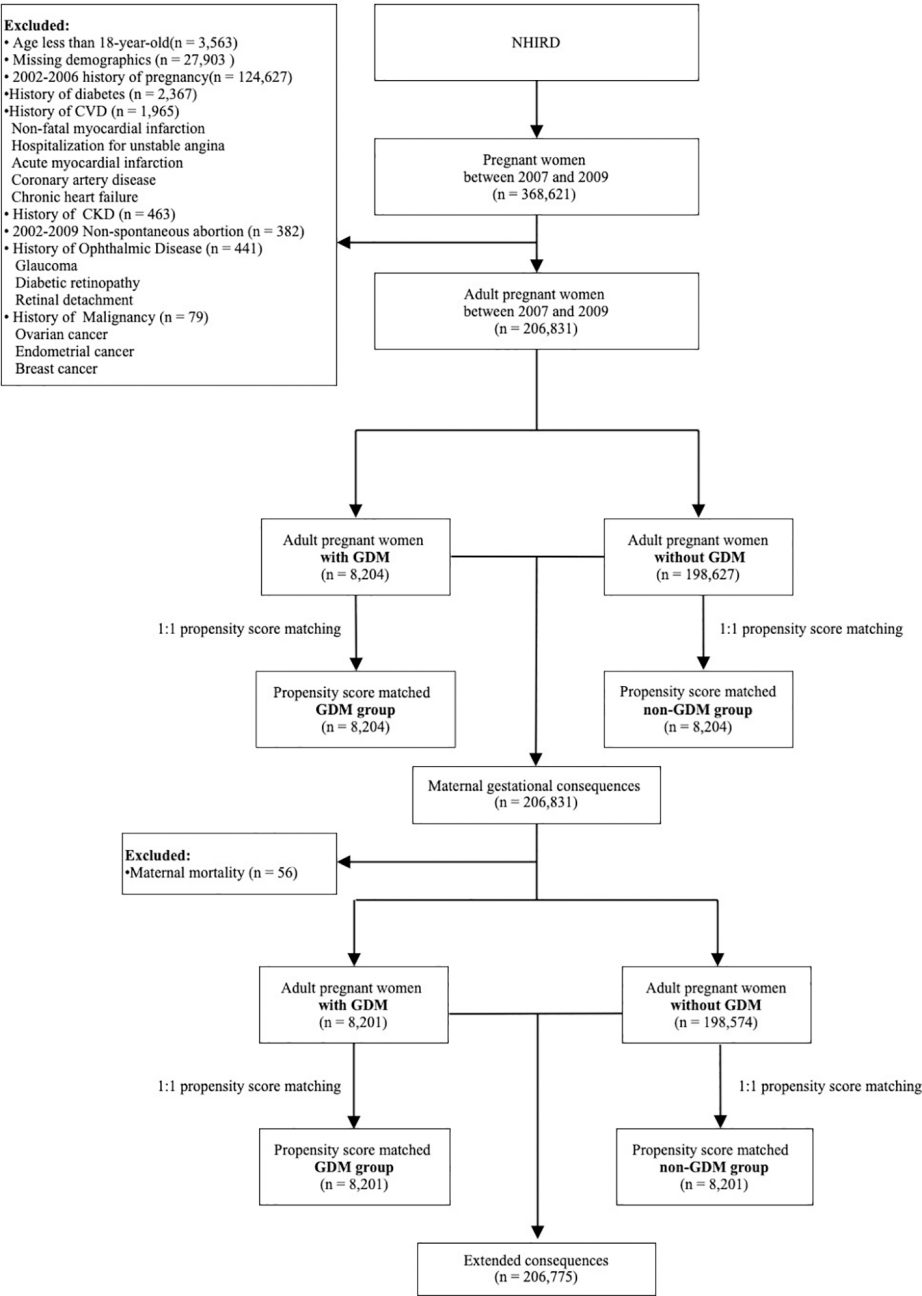
Flowchart of adult pregnant women inclusion and exclusion criteria for short-term (maternal gestational) and long-term (extended) consequences.

A total of 368,621 pregnant women between 1 January 2007 and 31 December 2009 were enrolled from the NHI Research Database. This research excluded the following criteria:age <18 years (n=3,563), missing demographics (n=27,903), history of pregnancy period of 2002–2006 (n=124,627), history of diabetes (n=2,367), history of CVD (n=1,965), history of CKD (n=463), non-spontaneous abortion (n=382) in 2002–2009, history of ophthalmic disease (n=441), and history of malignancy (n=79). Finally, 206,831 adult pregnant women were selected and divided into two groups with GDM (n=8,204) and without GDM (n=198,627). Maternal gestational consequences during pregnancy were analyzed among these patients. However, 56 patients died during this period, leaving 206,775 patients, who were finally divided into those with GDM (n=8,201) and without GDM (n=198,574), for the analysis of extended consequences. The selection of enrolled pregnant women and exclusion criteria are shown in **Figure 1**.

### Statistical analyses

The differences among pregnant women with GDM and without GDM were analyzed based on the demographic characteristics and history of the disease. The Chi-square test was used for categorical variables, which were expressed as n (%), and t-test was used for continuous variables, which were expressed as means ± standard deviation. To reduce baseline imbalance in measured characteristics, 1:1 propensity score matching was performed as age, endometriosis, pelvic inflammatory disease, PCOS, uterine polyps and uterine leiomyoma. Logistic regression analysis was performed to calculate the crude of odd ratio (OR) of short-term outcome events in the gestational period. Furthermore, Kaplan-Meier and Cox regression analyses were used for the incidence of type 2 DM, CKD, ophthalmic disease, CVD, and Malignancies. The results were expressed as hazard ratios (HRs) with 95% confidence intervals (CIs) for the incidence of long-term outcomes after delivery. Maternal age, endometriosis, pelvic inflammatory disease (PID), PCOS, uterine polyps, and uterine leiomyoma were included as covariates in the post-matching Cox regression models. Because the post-matching Cox models were fitted after propensity score matching on the measured baseline characteristics, including age, endometriosis, PID, PCOS, uterine polyps and uterine leiomyoma, these multivariable analyses were used to provide residual covariate adjustment and to improve estimate precision. The two groups on Kaplan-Meier were compared using log-rank test. An incidence rate is a ratio between a count and another measurement. The ratio of outcome events was based on the observed and the total number of person-years of observation (17). The difference between the two rates was analyzed. The *p*-value was obtained using the Chi^2^-statistic. A *P*-value less than 0.05 indicates that the two ratios are statistically significantly different. All analyses were performed using SAS, version 9.4 (SAS Institute, Cary, NC).

## Results

### Demographic and clinical characteristics of study population

**Table 1** shows the baseline characteristics of the study population for maternal gestational and extended consequences. A total of 206,831 adult pregnant women between 2007 and 2009 were finally enrolled in this study. The numbers of the adult non-GDM and GDM pregnant women were 198,627 and 8,204, respectively. Adult GDM pregnant women were older, with a mean age of 31.13 ± 4.47 years, had higher proportion of advanced maternal age (≥ 35 years old), and higher incidence of endometriosis, PCOS, uterine leiomyoma (all *p* < 0.001), PID (*p* = 0.024), and uterine polyps (*p* = 0.018) than non-GDM pregnant women. After 1:1 matching for age and comorbidities, as listed in **Table 1**, no significant difference was observed in the age and distribution of comorbidities between the two groups. During the pregnancy period, 56 pregnant women died. Therefore, the baseline numbers of adult pregnant women for extended consequences were 206,775. Among them, 198,574 were adult non-GDM pregnant women and the others were GDM pregnant women. Similarly, adult GDM pregnant women were old, had higher proportion of advanced maternal age (≥ 35 years old), and higher incidence of endometriosis, PCOS, uterine leiomyoma (all *p* < 0.001), PID (*p* = 0.026), and uterine polyps (*p* = 0.017) than non-GDM pregnant women. After 1:1 matching for age and comorbidities, no significant difference was observed in the age and distribution of comorbid between two groups.

**Table 1:** Demographics and clinical characteristics of adult pregnant women with and without gestational diabetic mellitus .

Baseline characteristics of adult pregnant women for analysis of maternal gestational consequences								
			Before matching			After 1:1 matching		
Variables		Total (n =206,831)	Non-GDM (n =198,627)	GDM (n=8,204)	*p*	Non-GDM (n=8,204)	GDM (n=8,204)	*p*
Age Group	< 35 year	179,803 (86.93)	173,356 (87.28)	6447 (78.58)	<0.001	6447 (78.58)	6447 (78.58)	1
	≥ 35 year	27,028 (13.06)	25,271 (12.72)	1757 (21.42)		1757 (21.42)	1757 (21.42)	
Age (mean ± sd)		29.49 ± 4.50	29.42 ± 4.50	31.13 ± 4.47	<0.001	31.13 ± 4.47	31.13 ± 4.47	1
Gynecological diseases								
Endometriosis		7360 (3.55)	7011 (3.53)	349 (4.25)	<0.001	340 (4.14)	349 (4.25)	0.726
PID		74,233 (35.89)	71,193 (35.84)	3040 (37.06)	0.024	3036 (37.01)	3040 (37.06)	0.948
PCOS		7050 (3.40)	6659 (3.35)	391 (4.77)	<0.001	391 (4.77)	391 (4.77)	1
Uterine polyps		1146 (0.55)	1085 (0.55)	61 (0.74)	0.018	65 (0.79)	61 (0.74)	0.720
Uterine leiomyoma		6141 (2.96)	5831 (2.94)	310 (3.78)	<0.001	282 (3.44)	310 (3.78)	0.230
Baseline characteristics of adult pregnant women for analysis of extended consequences								
			*Before Matching*			*After 1:1 Matching*		
Variables		Total (n =206,775)	Non-GDM (n=198,574)	GDM (n=8,201)	*p*	Non-GDM (n=8,201)	GDM (n=8,201)	*p*
Age Group	<35 year	179,758 (86.93)	173,313 (87.28)	6,445 (78.59)	<0.001	6,445 (78.59)	6,445 (78.59)	1
	≥35 year	27,017 (13.06)	25,261 (12.72)	1,756 (21.41)		1,757 (21.42)	1,757 (21.42)	
Age (mean ± sd)		29.49 ± 4.50	29.42 ± 4.50	31.13 ± 4.47	<0.001	31.13 ± 4.47	31.13 ± 4.47	1
Gynecological diseases								
Endometriosis		7,359 (3.55)	7,010 (3.53)	349 (4.26)	<0.001	348 (4.24)	349 (4.26)	0.969
PID		74,211 (35.88)	71,173 (35.84)	3,038 (37.04)	0.026	2,958 (36.07)	3,038 (37.04)	0.194
PCOS		7,049 (3.40)	6,658 (3.35)	391 (4.77)	<0.001	391 (4.77)	391 (4.77)	1
Uterine polyps		1,145 (0.55)	1,084 (0.55)	61 (0.74)	0.017	57 (0.7)	61 (0.74)	0.711
Uterine leiomyoma		6,139 (2.96)	5,830 (2.94)	309 (3.77)	<0.001	340 (4.15)	309 (3.77)	0.214

GDM, gestational diabetic mellitus; PID, pelvic inflammatory disease; PCOS, polycystic ovary syndrome; sd, standard deviation.

### Comparisons of maternal gestational consequences between the study groups

**Table 2** shows the maternal gestational and extended consequences between the pregnant women with or without GDM. In maternal gestational consequences, the incidence of preterm labor, preeclampsia, and gestational hypertension among adult pregnant women were significantly higher than those without GDM (all *p* < 0.001). After 1:1 matching for age and comorbidities, preterm labor, preeclampsia, and gestational hypertension remained significantly higher in adult pregnant women. **Table 3** shows the risk of maternal gestational and extended consequences between the pregnant women with or without GDM after 1:1 matching for age and comorbidities. Relative to adult non-GDM pregnant women, the crude ORs (cORs) of preterm labor, preeclampsia, and gestational hypertension were 1.72 (95% CI, 1.46–2.02), 2.84 (1.84–4.37), and 2.85 (1.52–5.37) in adult GDM pregnant women. After adjusting for age and comorbidities listed in **Table 1**, preterm labor, preeclampsia, and gestational hypertension remained significant risk factors in adult GDM pregnant women, with adjusted ORs (aORs) of 1.72, 2.86, and 2.85 (95% CI, 1.47–2.02, 1.86–4.40, and 1.53–5.41), respectively.

**Table 2:** Maternal gestational and the extended consequences among adult pregnant women with and without gestational diabetic mellitus .

Maternal gestational consequences							
Consequences	Total(n =206,831)	Before Matching			After 1:1 Matching		
		Non-GDM(n =198,627)	GDM(n=8,204)	*p*	Non-GDM(n=8,204)	GDM(n=8,204)	*p*
Pre-term	5,888(2.84)	5,476 (2.76)	412 (5.02)	<0.001	245 (2.99)	412 (5.02)	<0.001
Preeclampsia	700(0.33)	621 (0.31)	79 (0.96)	<0.001	28 (0.34)	79 (0.96)	<0.001
Gestational hypertension	288(0.13)	251 (0.13)	37 (0.45)	<0.001	13 (0.16)	37 (0.45)	<0.001
Extended consequences							
Consequences	Total (n =206,775)	Before Matching			*1:1 After Matching*		
		Non-GDM (n=198,574)	GDM (n=8,201)	*p*	Non-GDM (n=8,201)	GDM (n=8,201)	*p*
Type 2 DM	7,481(3.61)	6,588 (3.32)	893 (10.89)	<0.001	320 (3.90)	887 (10.82)	<0.001
CKD	2,018(0.97)	1,894 (0.95)	124 (1.51)	<0.001	77 (0.94)	118 (1.44)	0.003
Ophthalmic Disease	2,750(1.32)	2,544 (1.28)	206 (2.51)	<0.001	128 (1.56)	206 (2.51)	<0.001
CVD	6,336(3.06)	6,033 (3.04)	303 (3.69)	<0.001	262 (3.19)	295 (3.60)	0.154
Malignancies	1,753(0.84)	1,669 (0.84)	84 (1.02)	0.075	99 (1.21)	84 (1.02)	0.264

GDM, gestational diabetic mellitus; CKD, kidney disease; CVD, cardiovascular disease; Malignancies include ovarian or endometrial or breast cancer; Ophthalmic diseases include glaucoma or diabetic retinopathy or retinal detachmentchronic .

**Table 3:** Logistic regression and cox regression hazard model analysis of maternal gestational and the extended consequences of gestational diabetic mellitus.

Maternal gestational consequences					Extended consequences				
Events	Crude		Adjusted		Events	Crude		Adjusted	
	OR (95% CI)	*p*	OR (95% CI)	*p*		HR (95% CI)	*p*	HR (95% CI)	*p*
Pre-term	1.72 (1.46–2.02)	<0.001	1.72 (1.47–2.02)	<0.001	Type 2 DM	2.88 (2.53–3.27)	<0.001	2.88 (2.54–3.28)	<0.001
Preeclampsia	2.84 (1.84–4.37)	<0.001	2.86 (1.86–4.40)	<0.001	CKD	1.54 (1.15–2.05)	0.003	1.54 (1.15–2.05)	0.003
Gestational hypertension	2.85 (1.52–5.37)	0.001	2.87 (1.53–5.41)	0.001	Ophthalmic disease	1.62 (1.30–2.02)	<0.001	1.63 (1.30–2.03)	<0.001
					Cardiovascular disease	1.13 (0.95–1.33)	0.159	1.13 (0.95–1.33)	0.166
					Malignancies	0.85 (0.64–1.14)	0.274	0.85 (0.63–1.13)	0.266

CKD, chronic kidney disease; Ophthalmic diseases include glaucoma or diabetic retinopathy or retinal detachment; Malignancies include ovarian or endometrial or breast cancer; Adjusted with age, endometriosis, pelvic inflammatory disease, polycystic ovary syndrome, uterine polyps, and uterine leiomyoma.

### Comparisons of the extended consequences between the study groups

**Figure 2:**
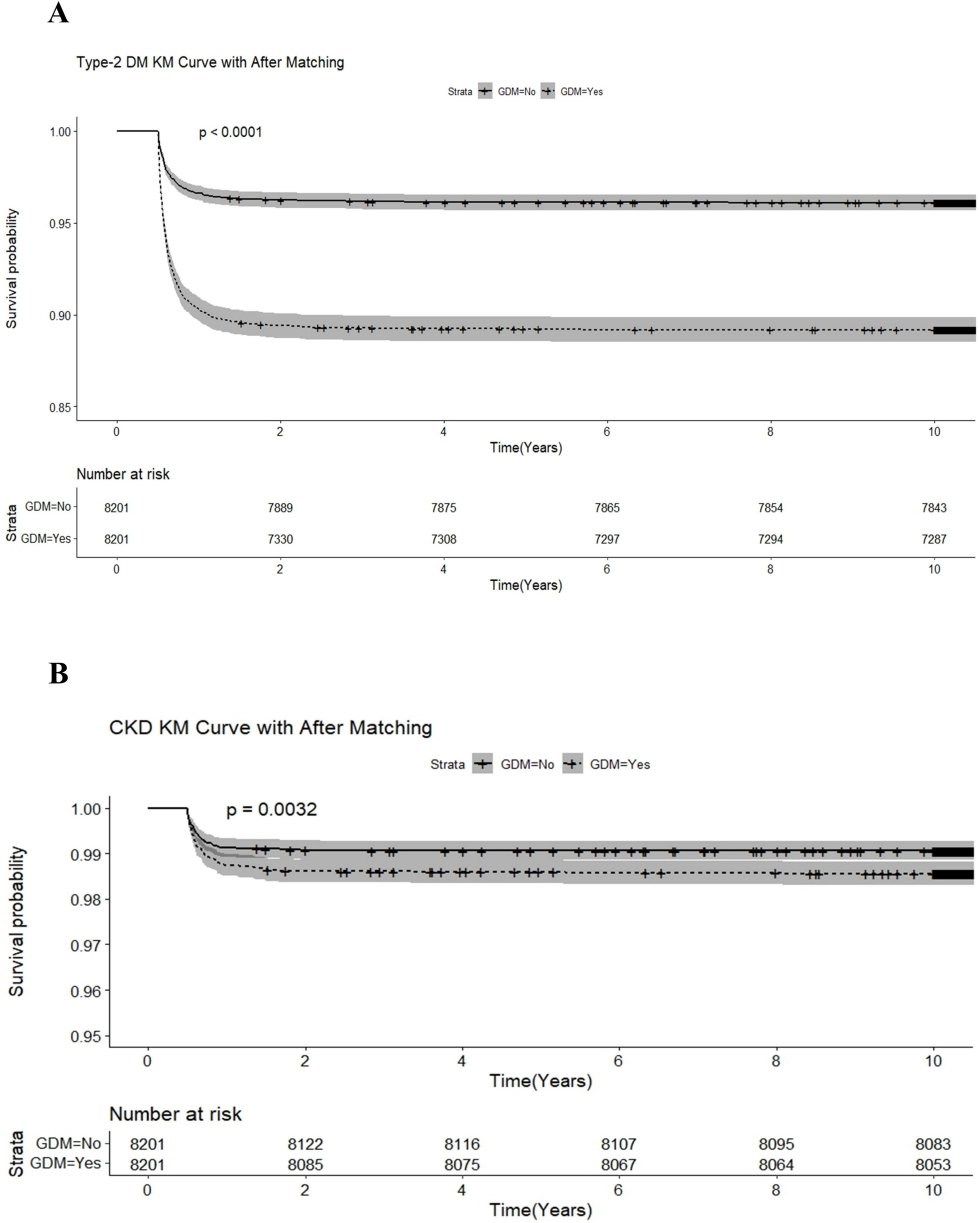
Kaplan-Meier analysis for the incidence of extended consequences among pregnant women with and without gestational diabetic mellitus. The extended consequences included **(A)** Type 2 DM **(B)** chronic kidney disease.

**Figure 3:**
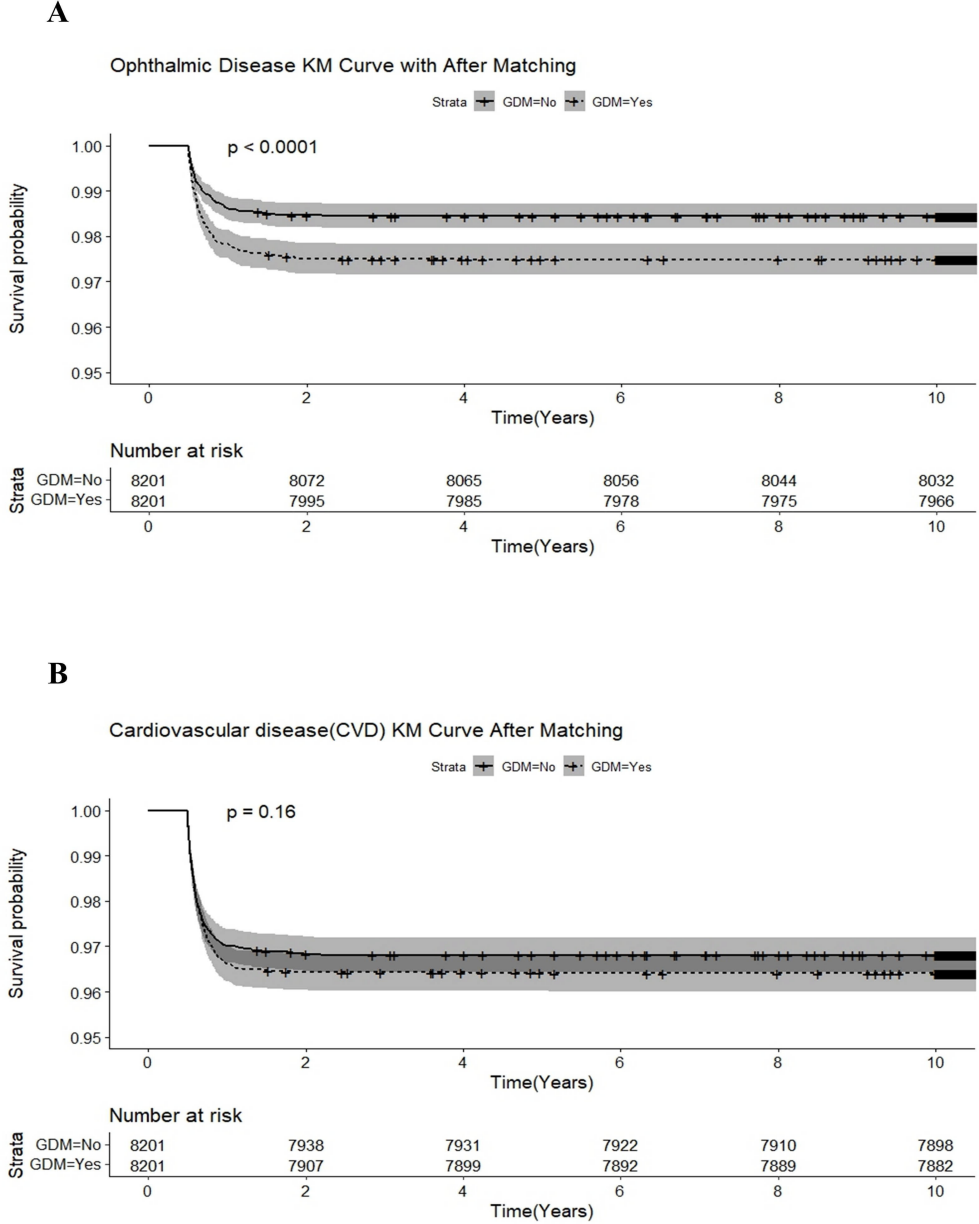
Kaplan-Meier analysis for the incidence of **(A)** ophthalmic disease and **(B)** cardiovascular disease among adult pregnant women with and without gestational diabetic mellitus.

**Figure 4:**
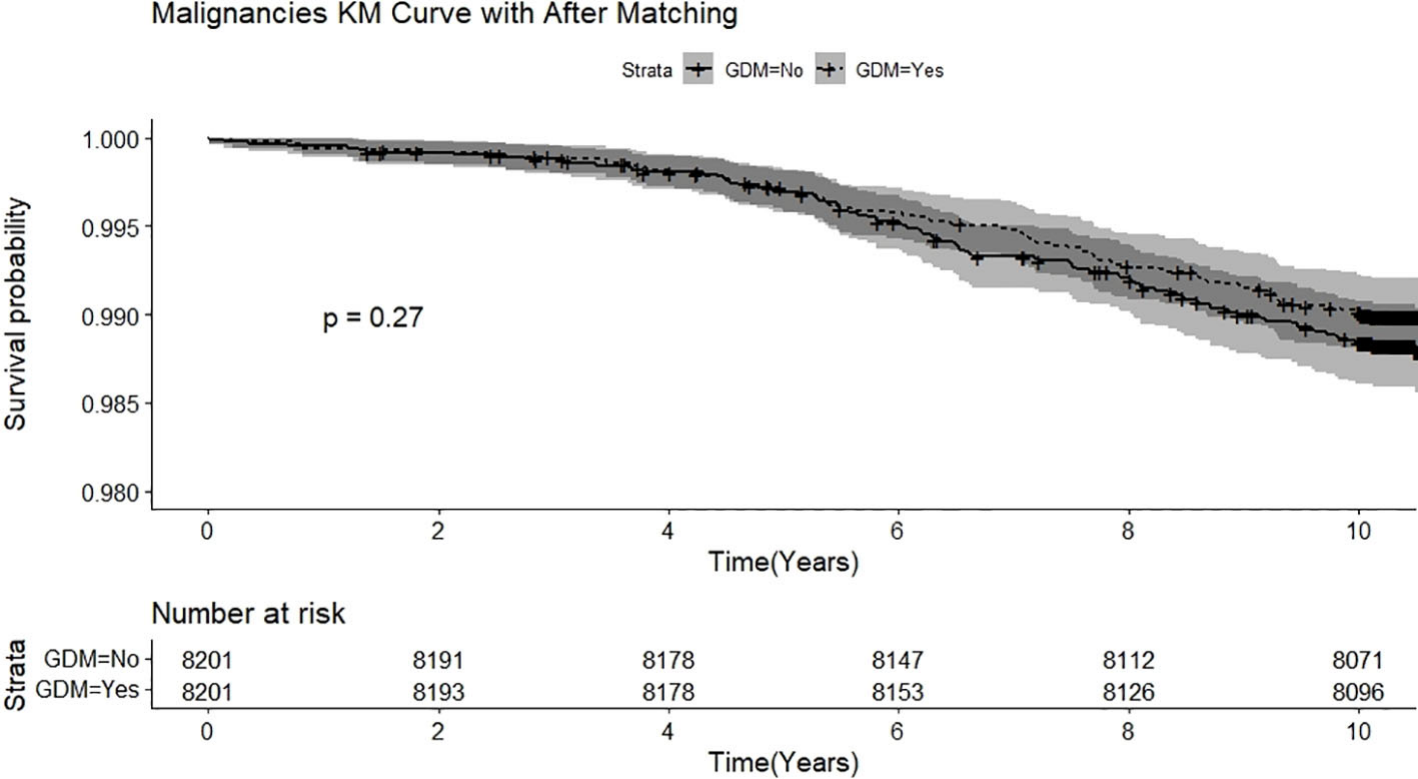
Kaplan-Meier analysis illustrating the incidence of malignancies among adult pregnant women with and without gestational diabetic mellitus.

In the extended consequences, the incidence of type 2 DM, cardiovascular disease, CKD, and ophthalmic disease among adult pregnant women were significantly higher than those without GDM (all *p* < 0.001). After 1:1 matching for age and comorbidities, the incidence of type 2 DM, CKD, and ophthalmic disease remained significantly high in adult pregnant women, as shown in **Table 2**. In **Table 3**, relative to adult non-GDM pregnant women, the crude HRs (cHRs) of type 2 DM, CKD and ophthalmic disease were 2.88 (95% CI, 2.53–3.27), 1.54 (1.15–2.05), and 1.62 (1.30– 2.02), respectively, in adult GDM pregnant women. After adjusting for age and comorbidities listed in **Table 1**, type 2 DM, CKD, and ophthalmic disease remained significant risk factors in adult GDM pregnant women, with adjusted HRs (aHRs) of 2.88, 1.54, and 1.63 (95% CI, 2.54–3.28, 1.15–2.05, and 1.30–2.03), respectively. During the follow-up period after delivery, Kaplan-Meier analysis significantly revealed lower incidence of type 2 DM (**Figure 2A**), CKD (**Figure 2B**), and ophthalmic disease (**Figure 3A**) among adult non-GDM pregnant women. The log-rank test was carried out at p values of *p* < 0.001, *p* = 0.003, and *p* < 0.001. No significant incidence of cardiovascular disease (**Figure 3B**) and malignancies (**Figure 4**) among adult pregnant women with and without GDM.

### Incident times to long-term event outcomes between the study groups

**Table 4** shows the comparison of incident times to long-term event outcomes between pregnant women with and without GDM. Relative to adult non-GDM pregnant women, the incidence of type 2 DM is significantly higher (*p* < 0.001) in the first 2 years postpartum among adult GDM pregnant women. For CKD and ophthalmic diseases, the incidence of both is significantly higher in the first year after delivery (*p* = 0.019; *p* < 0.001, respectively). However, this tendency was not observed after 2 years postpartum in type 2 DM, 1 year postpartum in CKD and ophthalmic diseases.

**Table 4:** Comparison of incident times of extended consequences among pregnant women with and without gestational diabetic mellitus.

Events	GDM			Non-GDM			p
	n	Total PY	Incidence	n	Total PY	Incidence	
Type 2 DM							
0y ≤ Follow up time < 1y	795	7,889	0.101	277	8,093	0.034	< 0.001
1y ≤ Follow up time < 2y	76	8,222	0.009	35	8,211	0.004	< 0.001
2y ≤ Follow up time < 10y	43	65,464	0.001	46	65,504	0.001	0.753
CKD							
0y ≤ Follow up time < 1y	103	8,161	0.013	72	8,173	0.008	0.019
1y ≤ Follow up time < 2y	13	8,206	0.001	7	8,240	0.001	0.180
2y ≤ Follow up time < 10y	32	65,543	0.0004	39	65,548	0.001	0.406
Ophthalmic disease							
0y ≤ Follow up time < 1y	178	8,132	0.022	113	8,159	0.014	< 0.001
1y ≤ Follow up time < 2y	28	8,210	0.003	16	8,206	0.002	0.071
2y ≤ Follow up time < 10y	29	65,543	0.0004	40	65,543	0.001	0.185
CVD							
0y ≤ Follow up time < 1y	278	8,097	0.034	245	8,105	0.030	0.146
1y ≤ Follow up time < 2y	16	8,205	0.002	18	8,209	0.002	0.732
2y ≤ Follow up time < 10y	25	65,552	0.0003	40	65,542	0.001	0.062
Malignancies							
0y ≤ Follow up time < 1y	5	8,198	0.0006	4	8,198	0.0004	0.739
1y ≤ Follow up time < 2y	3	8,202	0.0003	6	8,203	0.001	0.318
2y ≤ Follow up time < 10y	97	65,471	0.001	120	65,453	0.002	0.118

Follow-up for long-term outcomes was analyzed using time-to-event methods and interval-specific person-years, and the results should therefore be interpreted primarily through the hazard ratios and interval incidence summaries.

## Discussion

The study revealed significantly high incidence of preterm labor, preeclampsia, and gestational hypertension among adult pregnant women with GDM than those without GDM. In addition, the incidence of type 2 DM, CKD and ophthalmic disease among adult pregnant women were significantly higher than those without GDM during the follow-up. GDM remarkably affected the incidence of type 2 DM during the first 2 years after delivery. For CKD and ophthalmic diseases, GDM only had significant effect during first year postpartum. As an observational claims-based cohort study, the present analysis should be interpreted as estimating associations under routine clinical coding and follow-up conditions, rather than establishing definitive causal effects.

In the maternal gestational consequences, the presence of GDM was associated with remarkably increased incidence of preterm labor, preeclampsia, and gestational hypertension. A review article revealed that GDM have a slightly higher risk of spontaneous preterm labor than patients without carbohydrate metabolism disorders (relative risk [RR] 1.42; 95% CI 1.15-1.77) (11). Patients diagnosed with GDM have higher risk of developing gestational hypertension and preeclampsia (overall frequency 12%) compared with those without GDM (17, 18). The abovementioned studies are consistent with the results of increased risk of preterm labor, preeclampsia, and gestational hypertension among GDM women in Taiwan.

Many mechanisms can induce preterm birth among pregnant women with problems in carbohydrate metabolism, such as microvascular complication-related placental insufficiency and polyhydramnios induced by hyperglycemia. Hyperglycemia-induced immunosuppression can also increase the risk of opportunistic infection, thus increasing the risk of preterm birth (11). Gestational hypertension and preeclampsia are caused by oxidative stress, pro-inflammatory factor release, and vascular endothelial dysfunction. In the long-term follow-up, the incidence of type 2 DM, CKD, and ophthalmic diseases was higher among GDM patients in the study. The elevated hazard of subsequent type 2 DM in women with prior GDM was consistent with prior literature; however, the absolute cumulative incidence observed in this study should be interpreted as a claims-ascertained estimate under routine clinical care. Because the present study did not rely on protocol-based biochemical screening, the absolute incidence may be lower than that reported in cohorts with active postpartum surveillance. The molecular mechanism underlying chronic insulin resistance in GDM women persist 1 year postpartum (19). GDM also shares the same risk factors with type 2 DM such as obesity, Westernized diet, and family history of type 2 diabetes mellitus. These results have been widely documented (20–22). The incidence of CKD significantly increased among GDM patients (23). Renal insults during pregnancy such as gestational hypertension and preeclampsia, which are associated with endothelial dysfunction, can also lead to the development of CKD (24). This condition might be caused by the high incidence of type 2 DM related to the development of CKD. GDM-diagnosed women are only at heightened risk of CKD/ESKD if they develop type 2 DM in the years following pregnancy (25). The presence of GDM is associated with significantly higher incidence of ophthalmic morbidity such as glaucoma, retinal detachment, and diabetic retinopathy (26). In addition, the higher ophthalmic morbidity among GDM patients can be attributed to transient hyperglycemia related to small-vessel dysfunction such as retinal arteriolar abnormalities (27). In the present study, GDM remarkably affected type 2 DM in the first 2 years after episode of GDM. The result of follow-up for developing type 2 DM is roughly corresponding with a meta-analysis published on 2021 (28). This study shows that the incidence of type 2 DM after GDM is the highest within the first year after delivery. American Diabetes Association also recommends that woman who develops GDM during pregnancy should accept diabetes screening at 6–12 weeks postpartum. In comparison with the present study, in which the highest incidence of ophthalmic diseases was observed in 1 year postpartum, the incidence of ophthalmic diseases increases more steeply with age (26).

The incidence of cardiovascular diseases insignificantly increased in the GDM group in the study. The sample size may affect the results, because a large population of 1,515,079 pregnant patients showed that women with GDM have an elevated risk of cardiovascular outcomes, even in the absence of type 2 DM (29). The incidence of malignancies including endometrial, breast, or ovarian cancer did not increase in the study. For endometrial cancer, a median of 14 years between delivery and ascertainment of endometrial hyperplasia/endometrial cancer was set to estimate the association between GDM and endometrial cancer (30). The follow-up time in our study was very short to evaluate the outcome. A study enrolling 50,884 women aged 35–74 years showed that ever having GDM was not associated with breast cancer, and the other study with a mean follow-up duration of 15 years demonstrated no statistically significant associations between GDM and ovarian cancer, respectively (30). Although the risk of breast cancer is correlated to the frequency of individual GDM pregnancy, and an increased risk of ovarian cancer was observed in GDM women with gestational hypertension, our study was not designed to explore the subjects (30, 31).

This study has several limitations. First, individual-level body mass index, family history of GDM or diagnosis of type 2 DM or CKD by lab data were not available in the NHI Research Database, which might produce low outcome rates in the study. Second, the actual blood sugar level of patients cannot be collected from the NHI Research Database. The prognosis of individual is affected by how blood glucose level is controlled (32). In addition, gestational diabetes is determined using a consistent diagnostic method, such as 75-g Oral Glucose Tolerance Test (OGTT) or 100-g OGTT, which cannot be confirmed in the NHI Research Database. Third, whether all included women were truly primiparous could not be fully determined, although patients with a history of pregnancy from 2002 to 2006 were excluded. Fourth, only maternal information was obtained from the NHI Research Database, and it is not connected with the clinical status of her infants. Therefore, the infant outcomes of GDM, such as macrosomia, are hard to analyze. Finally, we observed the extended consequences with 10-year follow-up in the study. However, longer latency period for cancer might be necessary to evaluate the development of cardiovascular diseases and cancers (33). In addition, gestational diabetes is determined using a consistent diagnostic method, such as 75-g Oral Glucose Tolerance Test (OGTT) or 100-g OGTT, which cannot be confirmed in the NHI Research Database. Furthermore, because the study relied on claims-based diagnostic records rather than protocol-based laboratory screening, the absolute burden of postpartum dysglycemia or type 2 diabetes may have been underestimated when compared with studies using systematic laboratory assessment or structured follow-up (34).

This nationwide, population-based study showed that GDM was associated with higher risks of adverse pregnancy outcomes, including preterm labor, preeclampsia, cesarean delivery, and gestational hypertension, as well as higher subsequent risks of type 2 DM, CKD, and ophthalmic disease. Time-to-event analysis suggested that the excess incidence was concentrated in the early postpartum period, particularly for type 2 DM. These findings support closer postpartum surveillance after a pregnancy affected by GDM.

## Data Availability

The raw data supporting the conclusions of this article will be made available by the authors, without undue reservation.

## Supplementary material

Supplementary Table 1 Operational definitions and ICD-9-CM/ICD-10 codes for gestational diabetes mellitus, baseline comorbidities, and maternal outcome.s
