# Supplementary material for: Gestational and postpartum maternal consequences of gestational diabetes mellitus
Source: Front Endocrinol (Lausanne). 2026 Apr 15;17:1809244. doi: 10.3389/fendo.2026.1809244 (PMC13124484; doi:10.3389/fendo.2026.1809244)
Supplement: Supplementary Table 1 — Operational definitions and ICD-9-CM/ICD-10 codes for gestational diabetes mellitus, baseline comorbidities, and maternal outcome.s [file Table1.docx]

**Supplementary Table S1.** Operational definitions and ICD-9-CM/ICD-10 codes for gestational diabetes mellitus, baseline comorbidities, and maternal outcomes

|  | **Disease name** | **ICD-9-CM** | **ICD-10-CM** |
| --- | --- | --- | --- |
| Baseline | Pregnancy | 640-644, 645.1, 645.2, 646-648, 315.32, 650-677, 338.22 | O09.4, O09.5, O10-O16, O20-O29, O30-O48, O60-O77, O80-O82, O85-O92, O94-O9A, P61.0  EXCEPT:  O11.4, O11.5, O13.4, O13.5, O16.4, O16.5, O24.415, O24.425, O24.434, O24.435, O30.11-O30.19, O30.21-O30.29, O30.81-O30.89, O44.2-O44.5, O64.1-O64.3, O64.8, O65.0-O65.3, O65.8, O99.84 |
| Group | GDM | 648.81, 648.83, 648.84, V12.2 | O24.4, Z86.32 |
| Excluded | Pregancy with abortion | 632, 634, 637, 639, 640, 651.4, 651.5, 651.6, 651.3 | A34, O02.1, O03, O04, O08, O20.0, O31.1, Z33.2  EXCEPT:  O04.85-O04.88 |
|  | Spontaneous abortion | 634, 651.3, 651.4, 654.43, 654.5, 651.51, 651.53, 651.6 | O03, O06.38, O31.1 |
| Excluded/ Extended | Ovarian cancer | 183, 198.6, V10.43, V76.46, V16.41 | C56, C79.6, Z85.43, Z12.73 |
|  | endometrial cancer | 182 | C54.1 |
|  | breast cancer | 233, 174 | D05.00, D05.01, D05.02, D05.10, D05.11, D05.12, D05.80, D05.81, D05.82, D05.90, D05.91, D05.92, C50.011, C50.012, C50.019, C50.111, C50.112, C50.119, C50.211, C50.212, C50.219, C50.311, C50.312, C50.319, C50.411, C50.412, C50.419, C50.511, C50.512, C50.519, C50.611, C50.612, C50.619, C50.811, C50.812, C50.819, C50.911, C50.912, C50.919 |
|  | Glaucoma | 366.31, 365 | H26.23, H40 |
|  | Diabetic retinopathy | 250.5 | E08.31-E08.35, E09.31-E09.35, E11.31- E11.35 |
|  | Retinal detachment | 361 | H33 |
|  | Non-fatal MI | 410, 411 | I21.09, I22.0, I21.01, I21.02, I21.19, I22.1, I21.11, I21.29, I22.8, I21.4, I22.2, I21.21, I21.3, I22.9, I24.1 |
|  | AMI | 410, 429.79, 429.71, 429.5, 429.6, 411.81, 412 | I21, I22, I23, I24.0, I25.2 |
|  | Admission unstable angia | 413, 429.79 | I20.8, I20.1, I20.9, I23.7 |
|  | Coronary artery disease | 410, 401.11, 411, 412, 413, 414, 428 | I21.09, I22.0, I21.01, I21.02, I21.19, I22.1, I21.11, I21.29, I22.8, I21.4, I22.2, I21.21, I21.3, I22.9, I24.1, I20.0, I24.0, I24.8, I24.9, I25.2, I20.8, I20.1, I20.9, I25.1, I25.750, I25.751, I25.758, I25.759, I25.760, I25.764, I25.768, I25.769, I25.811, I25.812, I25.710, I25.711, I25.718, I25.719, I25.730, I25.731, I25.738, I25.739, I25.720, I25.721, I25.728, I25.729, I25.700, I25.701, I25.708, I25.709, I25.790, I25.791, I25.798, I25.799, I25.810, I25.3, I25.41, I25.42, I25.5, I25.6, I25.89, I25.9, I50.20, I50.21, I50.22, I50.23, I50.30, I50.31, I50.32, I50.33, I50.40, I50.41, I50.42, I50.43, I50.9, I50.1 |
|  | Chronic heart failure | 428 | I50.20, I50.21, I50.22, I50.23, I50.30, I50.31, I50.32, I50.33, I50.40, I50.41, I50.42, I50.43, I50.9, I50.1 |
|  | CKD | 585, 593.9 | N18.1-N18.6, N18.9 |
|  | Type 2 DM | 250, 251.8, 583.81, 366.41, 362.01, 362.02, 357.2 | E08.0, E08.3-E08.5, E08.641, E08.8, E09.01, E09.3-E09.5, E09.641, E09.8, E10.11, E10.2, E10.31, E10.36, E10.39, E10.4-E10.9, E11, E13.0-E13.5, E13.641, E13.8, E13.9  EXCEPT:  E08.37, E08.352-E08.355, E09.352-E09.355, E09.40, E10.42, E10.43, E13.10, E13.342-E13.345, E13.352-E13.355 |
| Gynecological diseases | Endometriosis | 617 | N80 |
|  | PID | 614.3-614.9, 616.9 | N73, N74 |
|  | Polycystic ovary syndrome | 256.4 | E28.2 |
|  | Uterine polyps | 621, 622.7 | N84 |
|  | Uterine leiomyoma | 218 | D25.0, D25.1, D25.2, D25.9 |
| Maternal gestational | Preterm | 644, 658.1, 658.2 | O42, O60 |
|  | Pre-eclampsia | 642.2, 642.4, 642.5, 642.7 | O11.1-O11.3, O11.9, O14.00-O14.03, O14.10-O14.13, O14.20-O14.23, O14.90-O14.93, O15.2 |
|  | Gestational hypertension | 642.3, 642.9 | O13.9, O13.1, O13.2, O13.3, O16.1, O16.2, O16.3, O16.9 |
